# St. Thomas's Hospital

**Published:** 1902-08-02

**Authors:** 


### ST. THOMAS'S HOSPITAL.

### THE YEAR'S WORK.

One thousand two hundred and thirty-one more individuals were treated in 1901 than in the previous year; of these, 784 were in-patients, and 331 accidents. These figures, the treasurer's report says, are strong evidence of the claims this hospital has upon the community, and of the great importance of the steps taken by the governors in deciding upon the large outlay now going on, both in the increase of operating theatres and casualty departments. It is hoped that the work will be completed before the end of the year. A new X-Ray and light department is in course of construction. The need of increasing the accommodation for the nursing staff has led to the suggestion that a rearrangement of the administrative block should be made, by which the house now occupied by the treasurer would be given to the nurses. By this means accommodation would be secured for about thirty nurses fully two years earlier than if a new building were waited for, and a covered way to and from the hospital would be available. A house would be provided for the treasurer at the river end of the block. The only drawback, says the report, appears to be the absorption of the

Governor's Hall, but as this is only used for the distribution of prizes on one afternoon in the year, it is thought that no real inconvenience would be caused. If the space between the present treasurer's house and the Stangate Road were utilised for a "proper Nurses' Home," it could be readily connected with the rearranged treasurer's house.

Efforts have been made to find a suitable site for a laundry, but unsuccessfully. A laundry would appear to be a real necessity, since 54,000 articles per month are used, and the present plan of sending these to a general laundry is unsatisfactory. A new system of Pasteurising the milk has been adopted, and an installation for cooling the larders and making ice has been ordered; with its aid it is hoped that the milk may be cooled after Pasteurising, and that the difficulty of preserving the milk during the summer months will disappear. The quantity of milk consumed in the year is 50,000 gallons.

The total number of beds in the hospital is 586. Of these 544 are at the service of the poor, while 42 are reserved for paying patients. The average daily number of patients during 1901 was 443 in the hospital, and 31·1 in St. Thomas's Home. The mean residence was 24·6 and 20·8 respectively. The total number treated in the wards (not including St. Thomas's Home) was 7,038. Of these, 5,974 were discharged, 596 died, and 468 remained at the end of the year. There were 17,804 out-patients, 42,698 casualties, 2,030 maternity patients, and 329 vaccinations. A total of 146,790 patients was seen during the year, or an average of 402 per day, while 2,030 women were attended at their own homes. The cases treated in the X-Ray department were: 309 in-patients, 421 out-patients, 44 special patients; and 1,176 skiagrams, etc., were taken.

The total ordinary income was £53,882 7s. 6d.; extraordinary, consisting of legacies and amount received from the Nightingale Fund, £11,206 7s. 4d. Annual subscriptions were £664 15s.; donations and boxes, £2,534 12s. 7d.; King Edward's Fund, £1,800; Sunday Fund, £959 2s. 4d., and £1,000 special donation; Saturday Fund, £183 2s. Invested property realised £46,264 16s. 7d.; patients' payments, £234 3s.; and £8,267 was realised on sale of stock on account of the new buildings.

The total ordinary expenditure was £55,501 13s. 2d.; extraordinary, repairs and reinstatements, £1,762 16s. 10d.; new operating theatres, children's wards, new casualty department and nurses' dining-rooms and other work, £17,828 15s. 3d.; miscellaneous, £347 16s. 4d. The sum of £33,459 16s. 6d. had been expended up to December 31st, 1901, on additions and improvements; a further sum of £20,000 is estimated as required to complete them. Liabilities in respect of tradesmen's accounts amount to about £12,000, £5,237 17s. 9d. of which will have to be provided out of the income for the current year.

A general court was held on June 11th, when an inspection of the hospital by the governors took place.

